# Multiple cellular compartments engagement in *Nicotiana benthamiana*-peanut stunt virus-satRNA interactions revealed by systems biology approach

**DOI:** 10.1007/s00299-021-02706-4

**Published:** 2021-05-24

**Authors:** Barbara Wrzesińska, Agnieszka Zmienko, Lam Dai Vu, Ive De Smet, Aleksandra Obrępalska-Stęplowska

**Affiliations:** 1 Department of Molecular Biology and Biotechnology, Institute of Plant Protection, National Research Institute, 20 Władysława Węgorka Street, 60-318 Poznan, Poland; 2 Institute of Bioorganic Chemistry, Polish Academy of Sciences, 12/14 Noskowskiego Street, 61-704 Poznan, Poland; 3 Faculty of Computing Science, Institute of Computing Science, Poznań University of Technology, 2 Piotrowo Street, 60-965 Poznan, Poland; 4 Department of Plant Biotechnology and Bioinformatics, Ghent University, Technologiepark 71, 9052 Ghent, Belgium; 5 VIB Center for Plant Systems Biology, Technologiepark 71, 9052 Ghent, Belgium

**Keywords:** Virus infection, Peanut stunt virus, Plant–virus interactions, Phosphorylation, Small RNAs, Defense response

## Abstract

**Key message:**

PSV infection changed the abundance of host plant’s transcripts and proteins associated with various cellular compartments, including ribosomes, chloroplasts, mitochondria, the nucleus and cytosol, affecting photosynthesis, translation, transcription, and splicing.

**Abstract:**

Virus infection is a process resulting in numerous molecular, cellular, and physiological changes, a wide range of which can be analyzed due to development of many high-throughput techniques. Plant RNA viruses are known to replicate in the cytoplasm; however, the roles of chloroplasts and other cellular structures in the viral replication cycle and in plant antiviral defense have been recently emphasized. Therefore, the aim of this study was to analyze the small RNAs, transcripts, proteins, and phosphoproteins affected during peanut stunt virus strain P (PSV-P)–*Nicotiana benthamiana* interactions with or without satellite RNA (satRNA) in the context of their cellular localization or functional connections with particular cellular compartments to elucidate the compartments most affected during pathogenesis at the early stages of infection. Moreover, the processes associated with particular cell compartments were determined. The ‘omic’ results were subjected to comparative data analyses. Transcriptomic and small RNA (sRNA)–seq data were obtained to provide new insights into PSV-P–satRNA–plant interactions, whereas previously obtained proteomic and phosphoproteomic data were used to broaden the analysis to terms associated with cellular compartments affected by virus infection. Based on the collected results, infection with PSV-P contributed to changes in the abundance of transcripts and proteins associated with various cellular compartments, including ribosomes, chloroplasts, mitochondria, the nucleus and the cytosol, and the most affected processes were photosynthesis, translation, transcription, and mRNA splicing. Furthermore, sRNA-seq and phosphoproteomic analyses indicated that kinase regulation resulted in decreases in phosphorylation levels. The kinases were associated with the membrane, cytoplasm, and nucleus components.

**Supplementary Information:**

The online version contains supplementary material available at 10.1007/s00299-021-02706-4.

## Introduction

The presence of a virus in a host cell interferes with host cell metabolism and utilizes cell structural elements, including membranous ones, for its multiplication and cell-to-cell movement. For their replication, viruses can use host proteins, membranes, lipids and metabolites to assemble viral replication complexes (VRCs), also called viral factories, on subcellular membrane surfaces. They may exploit the membranes of organelles such as the endoplasmic reticulum (ER), mitochondria, vacuoles, Golgi apparatus, chloroplasts, peroxisomes, or plasma membrane (Nagy and Pogany 2012). Afterwards, plant viruses move from an infected cell to its neighboring cells through plasmodesmata (PDs) that are modified by viral movement proteins (MPs). Before that, the viruses are moved from viral factories to PDs by host cytoskeletal elements (Schoelz et al. 2011; Ueki and Citovsky 2011). It has also been reported that some plant viruses may hijack the host secretory pathway and protein transport machineries for efficient transport to PDs and for cell-to-cell movement (Patarroyo et al. 2013). The presence of plant viruses not only alters the levels of proteins but also changes the levels of protein posttranslational modifications (e.g., phosphorylation) and host gene transcripts, as shown for peanut stunt virus (PSV), in host cells (Obrępalska-Stęplowska et al. 2013, 2018; Wrzesińska et al. 2018). In addition, host plants dealing with cellular infection enter a new state as a result of the triggering of response reactions. One such defense reaction is posttranscriptional gene silencing (PTGS), which is also a crucial factor in the regulation of gene expression during development and genome stability maintenance. Plants utilize PTGS to efficiently and specifically recognize and eliminate invading viruses (Baulcombe 2004; Wieczorek and Obrępalska-Stęplowska 2015).

PTGS is triggered by small RNAs (sRNAs) that are divided into two main classes: microRNAs (miRNAs) and short interfering RNAs (siRNAs). Primary miRNAs are transcribed from *MIR* genes. These primary miRNAs form stem-loop structures to create double-stranded RNAs (dsRNAs), which are processed by DICER-like 1 (DCL1) to yield a mature miRNAs of approximately 21–24 nt. These mature miRNAs are incorporated into Argonaute 1 (AGO1) to form an RNA-induced silencing complex (RISC), which directs the cleavage of mRNAs (Voinnet 2009). Additionally, miRNAs can target other noncoding RNAs to generate siRNAs referred to as trans-acting siRNAs (ta-siRNAs). ta-siRNAs are transcribed from *TAS* genes and are then recognized and cleaved by RISC coupled with particular miRNAs. Then, RNA-dependent RNA polymerase (RDR6) uses the cleaved products to produce dsRNAs, which are sequentially cleaved by DCL proteins to produce phased 20–24 nt ta-siRNAs (Allen et al. 2005; Zhang et al. 2012).

PSV belongs to the *Cucumovirus* genus (*Bromoviridae* family), which is a virus genus distributed worldwide. PSV infects plants belonging to Fabaceae family, e.g., *Lupinus luteus*, *Phaseolus vulgaris*, as well as Solanaceae family, including genera such as *Nicotiana* (e.g., *Nicotiana benthamiana*), *Datura* (e.g., *Datura stramonium*), and *Solanum* (e.g., *Solanum lycopersicum*) (Bananej et al. 1998; Obrępalska-Stęplowska et al. 2008b). The PSV genome consists of three positive-sense single-stranded RNA [( +)ssRNA] genomic RNAs and two subgenomic RNAs. RNA1 encodes the 1a protein, which together with the 2a protein synthesized from RNA2 constitutes the VRC. RNA2 also has an open reading frame encoding the 2b protein, which is synthesized from the subgenomic RNA RNA4A and is known to participate in viral movement and silencing suppression (Ding et al. 1994; Netsu et al. 2008). RNA3 is bicistronic and encodes an MP (3a) and a coat protein (CP) (which is synthesized from subgenomic RNA4) (Mushegian and Koonin 1993). Moreover, some PSV strains, e.g., PSV-P, may include a noncoding small ( +)ssRNA particle, satellite RNA (satRNA), that does not encode functional proteins and requires a helper virus for its replication (Obrępalska-Stęplowska et al. 2008a; Simon et al. 2004). SatRNAs are able to change the levels to which helper viruses accumulate (usually decreasing them) and the severity of infection (usually attenuating it), depending on the strain of the helper virus, the sequence of the satRNA, the host, and the environmental conditions (Liao et al. 2007; Obrępalska-Stęplowska et al. 2015; Simon et al. 2004). It has been shown that satRNA-P can cause different plant response in association with two different PSV strains under various temperature conditions in *N. benthamiana* plants (Obrępalska-Stęplowska et al. 2015, 2018).

Most high-throughput analyses of plant responses to virus infections have mainly focused on biological processes affected by pathogenesis; however, proteins, transcripts, or transcripts targeted by specific miRNAs associated with cellular compartments have been less frequently analyzed. Therefore, sRNA (miRNA and ta-siRNA) analysis together with transcriptomic analysis was performed to investigate the processes and the regulation of gene expression, associated with specific cellular compartments. Moreover, our previous studies on the influence of PSV-P and satRNA-P on the *N. benthamiana* proteome and phosphoproteome not only revealed changes in photosynthesis and carbon metabolism, observed in virus-infected plants, but also the alterations in the level of proteins involved in RNA synthesis, transport, and turnover (Wrzesińska et al. 2018). Strong downregulation of overall protein phosphorylation in virus-treated plants (without satRNA) was reported. Therefore, in this study, we aimed to complement the previously obtained proteomic and phosphoproteomic data with the analysis of cellular compartments affected during the early stages of PSV-P and PSV-P + satRNA-P infection in *N. benthamiana* plants. Based on the collective results, we concluded that the most profoundly affected cellular compartments during PSV-P and PSV-P + satRNA infection in *N. benthamiana* plants are ribosomes, the cytosol, chloroplasts, mitochondria, and the nucleus.

## Materials and methods

### Plant and virus materials

*N. benthamiana* plants and PSV-P with or without satRNA-P inoculum were prepared for the experiments as described previously [for sRNA analyses: according to Wrzesińska et al. (2018), for transcriptomic analyses: according to Obrępalska-Stęplowska et al. (2018)]. Briefly, infectious copies of PSV-P and satRNA were synthetized as described previously (Obrępalska-Stęplowska et al. 2013). Four-leaf-stage *N. benthamiana* seedlings were inoculated with infectious clones of PSV-P or PSV-P + satRNA or with inoculation buffer alone (negative control). *N. benthamiana* biological replicates were subjected to high-throughput analyses; four were used for sRNA analysis, and three were used for transcriptomic and (phospho)proteomic analyses. *N. benthamiana* leaves above the inoculated leaves were harvested at 5 days post inoculation (dpi) for (phospho)proteomic and sRNA analyses, whereas they were harvested at 20 dpi for transcriptomic analysis. To validate the resulting data, the plant material was prepared as described above.

### sRNA analysis

Total RNA from the harvested plant material was extracted as described previously (Wieczorek et al. 2013) and subjected to DNA digestion using RNase-free DNase I (Thermo Fisher Scientific, Waltham, MA, USA) according to the manufacturer’s instructions.

To analyze viral genomic RNA levels, 500 ng of digested RNA was subjected to reverse transcription using RevertAid Reverse Transcriptase (Thermo Fisher Scientific) with a random hexamer primer (Thermo Fisher Scientific). PSV-P and satRNA-P were detected by quantitative real-time PCR (RT-qPCR). The reactions were completed in a Mx3500P thermal cycler (Agilent Technologies, Santa Clara, CA, USA). The reaction was conducted in a 10 µL solution using iTaq Universal SYBR Green Supermix (Bio-Rad, Hercules, CA, USA) with 0.5 µM forward and reverse primers (PSVq1, PSVq2b, PSVqCP, PARNA, and NbAct (Wrzesińska et al. 2018) and 1 μL of cDNA. The reaction profile consisted of an initial denaturation step at 95 °C for 3 min followed by 40 cycles of 95 °C for 20 s, an annealing step for 20 s (at the temperatures listed in the Supplementary materials of a previous publication (Wrzesińska et al. 2018) and 72 °C for 20 s. Dissociation curves were generated from 70 to 95 °C. Based on their highest RNA integrity number (RIN) scores and similar PSV-P genomic RNAs accumulation levels, four samples were used for subsequent analysis. sRNA next-generation sequencing (NGS) was performed on an Illumina platform (Genomed S.A., Warsaw, Poland). The resulting sequencing data were deposited into the Gene Expression Omnibus with the dataset identifier GSE128200.

The obtained sRNA-seq data were preprocessed. Briefly, adaptor-free sRNA reads were subjected to quality filtering with fastq_quality_filter from the FASTX-Toolkit package (http://hannonlab.cshl.edu/fastx_toolkit/) using the -p 95 and -q 20 parameters. Then, the sequence redundancy and read counts (i.e., raw expression values) were determined with fastx_collapser from the same package.

For basic annotation, short reads were compared against noncoding RNAs from Rfam (Kalvari et al. 2018) and miRNA precursors from miRBase (Kozomara and Griffiths-Jones 2014). The comparison was performed with Bowtie (Langmead et al. 2009), with no mismatches allowed.

miRNAs and ta-siRNAs were identified using ShortStack (Axtell 2013). As a reference, the *N. benthamiana* genome file Nbv0.5.genome.fa (Naim et al. 2012) was used.

To pinpoint the differentially expressed sRNAs, the DESeq2 package (Love et al. 2014) was applied.

To identify sRNA targets, the psRNATarget tool was used (Dai and Zhao 2011) with the default settings. The miRNA targets were searched among transcripts from the file Nbv5.1_transcriptome_primary_correct.fa (Nakasugi et al. 2014). Additionally, miRNA and ta-siRNAs associations with genomic RNAs of PSV-P (GenBank accession numbers EU570236.1, EU570237.1, and EU570238.1), PSV-G (GenBank accession numbers JN135294.1, JN135293.1, and JN135292.1), PSV-Ag (GenBank accession numbers GU126412.1, GU129698.1, and JF897622.1), satRNA-P (GenBank accession number EF535259.1), and satRNA-Ag (GenBank accession number EF469733.1) were investigated. PSV-specific sRNAs (miRNAs and ta-siRNAs) targeting *N. benthamiana* transcript sequences were also identified.

Furthermore, to assign GO terms to *N. benthamiana* transcripts, transcriptome annotation was performed with Trinotate v 3.0.2 (https://trinotate.github.io/). First, a search against the Swiss-Prot database, a nonredundant and manually curated set of proteins from the UniProt database (http://www.uniprot.org/), was conducted. For this search, BLASTX from the BLAST package with the -max_target_seqs 1 option was used. Then, the open reading frames were predicted with TransDecoder v 5.0.1 (https://github.com/TransDecoder/TransDecoder/releases/tag/v5.0.1), and the predicted protein sequences were searched against Swiss-Prot using BLASTP from the BLAST package (option: -max_target_seqs 1). Next, the transcriptomes were searched against protein domains from the Pfam database (http://pfam.xfam.org/) using hmmscan (hmmer.org) with the default settings. Finally, the above search results were loaded into an SQLite database of Trinotate, and a report was generated with the report tool in Trinotate. Thereafter, psRNATarget and Trinotate output data were subjected to analysis with goseq software (Young et al. 2010). All the targeted transcripts served as the background for the enrichment analysis. Additionally, GO enrichment analysis of PSV-P-targeting sRNAs targeting *N. benthamiana* transcripts was performed using Blast2GO Pro software.

### Transcriptomic analysis

The following procedures for transcriptomic analyses of *N. benthamiana* plants infected with PSV-P with or without satRNA-P and of control plants were performed as described previously (Obrępalska-Stęplowska et al. 2018): RNA extraction and detection of viral and satRNA in the plants, transcriptome profiling with species-specific microarrays, data acquisition, and statistical analysis. The resulting transcriptome data of the PSV-P- and PSV-P + satRNA-infected *N. benthamiana* were deposited in the Gene Expression Omnibus with the dataset identifier GSE133124, whereas the transcriptome data of the mock-inoculated plants (which were also used as controls in transcriptomic analyses of PSV-G-infected plants (Obrępalska-Stęplowska et al. 2018)) were previously deposited under accession number GSE104026.

Functional annotation of the differentially expressed genes (DEGs) during PSV-P or PSV-P + satRNA infection and GO enrichment analysis were performed using Blast2GO Pro software with the default settings. The transcript sequences of all identified genes were input into Blast2GO Pro software and blasted against the NCBI nr protein sequence database of green plants (Viridiplantae) with the default settings. Subsequently, the results were examined for GO annotation and then subjected to Fisher’s exact test (p < 0.05) to extract the enriched GO terms.

### (Phospho)proteomic analysis

(Phospho)proteomic data associated with PSV-P and PSV-P + satRNA pathogenesis processes were acquired from a previous publication (Wrzesińska et al. 2018). In this study, the data were analyzed for the affected cellular components of *N. benthamiana* plants infected with PSV-P and PSV-P + satRNA-P, which was not the aim of the previous study. Gene Ontology (GO) enrichment analysis of cellular components was performed using Blast2GO Pro software (Götz et al. 2008) with the default settings. The proteins and phosphoproteins for which the levels (normalized to the total protein levels) were significantly different between samples (two-sample test, *p* value < 0.05) were identified, and the unique proteins and phosphoproteins that were found in all replicates of the mock, PSV-P or PSV-P + satRNA samples were used for further analysis. The protein sequences of all identified proteins and phosphoproteins were input into Blast2GO Pro software and blasted against the NCBI nr protein sequence database of green plants (Viridiplantae) with the default settings. Subsequently, the results were examined for GO annotation and subjected to Fisher’s exact test (*p* < 0.05) to extract the enriched GO terms.

### Data integration

To integrate results of our analyses, we used three databases: proteomic (SolGenomics database (Fernandez-Pozo et al. 2014)) and two transcriptomic [Nb-105 k microarray based on transcriptome v.3—v.5, and v5.1 Nakasugi et al. 2013, 2014)]. To analyze potential protein–protein interactions, Nb-105 k microarray was aligned to the transcriptome v5.1 using OmicsBox software. Afterwards, Nb-105 k microarray and transcriptome v5.1 databases accession numbers were confronted with the corresponding SolGenomics accession numbers [the data has been provided by Peter Waterhouse's laboratory (www.benthgenome.qut.edu.au)].

KAAS, the KEGG Automatic Annotation Server (Moriya et al. 2007), was used to classify differently regulated proteins, DEGs and target transcripts of differentially expressed miRNAs in PSV-P- and PSV-P + satRNA-infected *N. benthamiana* into appropriate pathways. miRNA targets and DEGs or differentially regulated proteins classified to the same pathways were selected and the corresponding protein sequences served as an input for protein–protein interaction networks analysis using STRING (https://stringdb.org) (Szklarczyk et al. 2019). *S. tuberosum* was set as the reference organism. The minimum required interaction score was set as high confidence (> 0.700) and the active interaction sources were chosen as follows: text mining, experiments, databases, co-expression, gene fusion, and co-occurrence. The results were visualized using Cytoscape (version 3.6.0) (Shannon et al. 2003) with *S. tuberosum* ID numbers converted to *N. benthamiana* ID numbers from SolGenomics database.

### RT-qPCR

To validate transcriptomic data, 3 µg of total RNA extracted from plants harvested at 20 dpi (for DEG validation) or 2 µg of total RNA extracted from plants harvested at 5 dpi (for kinase transcripts validation) was used in reverse transcription reaction performed with Maxima First Strand cDNA Synthesis Kit for RT-qPCR, with dsDNase [Thermo Fisher Scientific). The resulting cDNA mixture was diluted with 20 µL of H_2_O. Gene expression was analyzed by RT-qPCR using iTaq Universal SYBR Green Supermix (Bio-Rad) with 0.5 µM forward and reverse primers (Obrępalska-Stęplowska et al. (2018) and Table S1] and 1 µL of appropriate cDNA, in 10-µL reaction, in a QuantStudio 5 Real-Time PCR System (Applied Biosystems, Waltham, Massachusetts, USA). For DEG analysis, the reaction profile consisted of an initial denaturation step at 95 °C for 3 min, followed by 40 cycles of 95 °C for 20 s, and annealing and elongation steps at 60 °C for 1 min. For kinase transcript analysis, the reaction profile consisted of an initial denaturation step at 95 °C for 3 min, followed by 40 cycles of 95 °C for 20 s, an annealing step for 30 s (at the temperatures listed in Table S1), and an elongation step at 72 °C for 30 s. Dissociation curves were generated from 65 to 95 °C. Tested genes were normalized to *EF1a* and *β-actin* genes (Wrzesińska et al. 2018). Data were analyzed using GenEx version 6 (Multid Analyses AB, Göteborg, Sweden). The comparison with t-test of the expression of all tested genes between two groups—PSV-P or PSV-P + satRNA versus control was performed. P values lower than 0.05 indicated statistically significant results.

To validate miRNA, a real-time stem-looped RT-qPCR procedure was applied. Total RNA extracted from plants harvested at 5 dpi was DNase I (Thermo Fisher Scientific) treated and purified using TriReagent solution (Thermo Fisher Scientific). DNase-treated RNA samples were diluted to a final concentration of 10 ng/µL. Stem-loop reverse transcription primers were designed according to Baksa et al. (2015), while the reference gene—small nucleolar RNA U6 primers sequences were based on Turner et al. (2013) (Table S2). Stem-loop pulsed reverse transcription was done according to Varkonyi-Gasic and Hellens (2011). Twenty µL reaction contained the following components: 0.5 µL (10 mM) dNTP mix, 1 µL (1 µM) denatured U6 stem-loop RT primer, 1 µL (1 µM) denatured miRNA stem-loop RT primer, 4 µL 5X First-Strand buffer, 2 µL (0.1 M) DTT, 4 U RiboLock (Thermo Fisher Scientific), 50 U SuperScript™ III Reverse Transcriptase (Thermo Fisher Scientific), and 2 µL RNA template. No RT reactions were prepared as the controls. The reactions were performed in Mastercycler nexus (Eppendorf, Hamburg, Germany): incubation at 16 °C for 30 min followed by 60 cycles at 30 °C for 30 s, 42 °C for 30 s, and 50 °C for 1 s, and enzyme inactivation at 85 °C for 5 min. RT-qPCR reaction was performed in 20 µL volume using iTaq Universal SYBR Green Supermix (Bio-Rad) with 0.5 µM forward and 0.5 µM universal reverse primers (Table S2), and 2 µL of cDNA. The reactions were completed in a QuantStudio 5 Real-Time PCR System (Applied Biosystems). The reaction profile consisted of initial denaturation step at 95 °C for 5 min, followed by 40 cycles of 95 °C for 5 s, and 60 °C for 10 s. Dissociation curves were generated from 65 to 95 °C at 0.2 °C. The results were analyzed using GenEx version 6 (Multid Analyses AB, Göteborg, Sweden). The comparison with t-test of the expression of tested miRNAs between two groups—PSV-P or PSV-P + satRNA versus control was performed. P values lower than 0.05 indicated statistically significant results.

## Results

### sRNA profiling of *N. benthamiana* infected with PSV-P (and satRNA) indicate changes in miRNAs and ta-siRNAs associated mainly with the nucleus and cytoskeleton

sRNA sequencing of *N. benthamiana* infected with PSV-P and PSV-P + satRNA revealed that the majority of sRNAs belonged to the 24 nt size class followed by the 20 nt size class (Fig. S1).

A search for miRNAs performed using ShortStack (Axtell 2013) yielded 143 mature miRNAs. However, a comparison of these miRNAs to known miRNAs (plant miRNAs from miRBase, release 21) allowing for up to 5 mismatches and no gaps resulted in 41 records, suggesting that a large fraction of the discovered miRNAs were novel; their identification might have been correct but was unexpected. Therefore, the known mature miRNAs identified in *Viridiplantae* were searched in the sequencing data, resulting in 243 sequences. This finding suggested that a substantial portion of true miRNAs was missing from the ShortStack results. Consequently, less strict criteria for the miRNA search (different from the default settings) were used; for example, candidates with no miRNA* sequence were accepted. This approach yielded 2539 mature miRNAs, which was more than expected for a plant species. Analysis of the intersection of the 2539 identified miRNAs with known miRNAs resulted in a final list of 185 miRNA candidates (Table S3A).

ShortStack was also applied to identify ta-siRNAs. To obtain the highest quality set of these sRNAs, only the top 200 candidates from each sample were selected based on the ‘phased score value’ provided by ShortStack. Finally, the candidate sRNAs from all samples were merged into a single dataset of nonredundant phased siRNAs containing 320 sequences (Table S3B).

Differential expression analyses performed with the DESeq2 package resulted in few differentially expressed miRNAs and ta-siRNA in the two test conditions compared to the control condition (Table 1A). The levels of two miRNAs changed in both test conditions, while the levels of only one ta-siRNA changed in plants treated with PSV-P + satRNA.

**Table 1:**
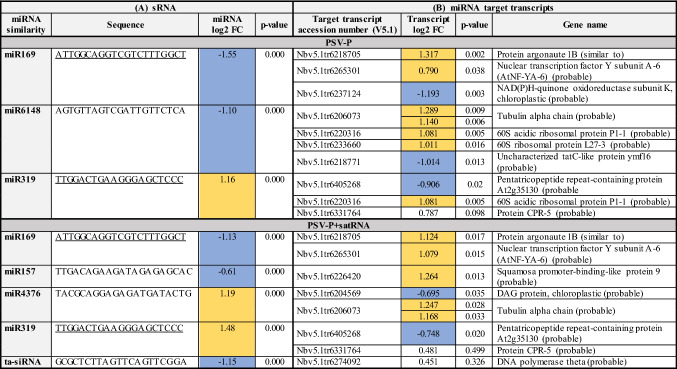
(A) Differentially expressed miRNAs and ta-siRNAs in PSV-P- and PSV-P + satRNA-treated *N. benthamiana*. (B) Potential miRNA target transcripts found within differentially expressed genes obtained from transcriptomic data analyses

The values in colored cells were statistically significant (*p* value < 0.05). The boxes are color-marked depending on the direction of the expression change direction: yellow indicates upregulation, and blue indicates downregulation. The underlined sequences are present in both conditions

*FC* fold change

**Fig. 1:**
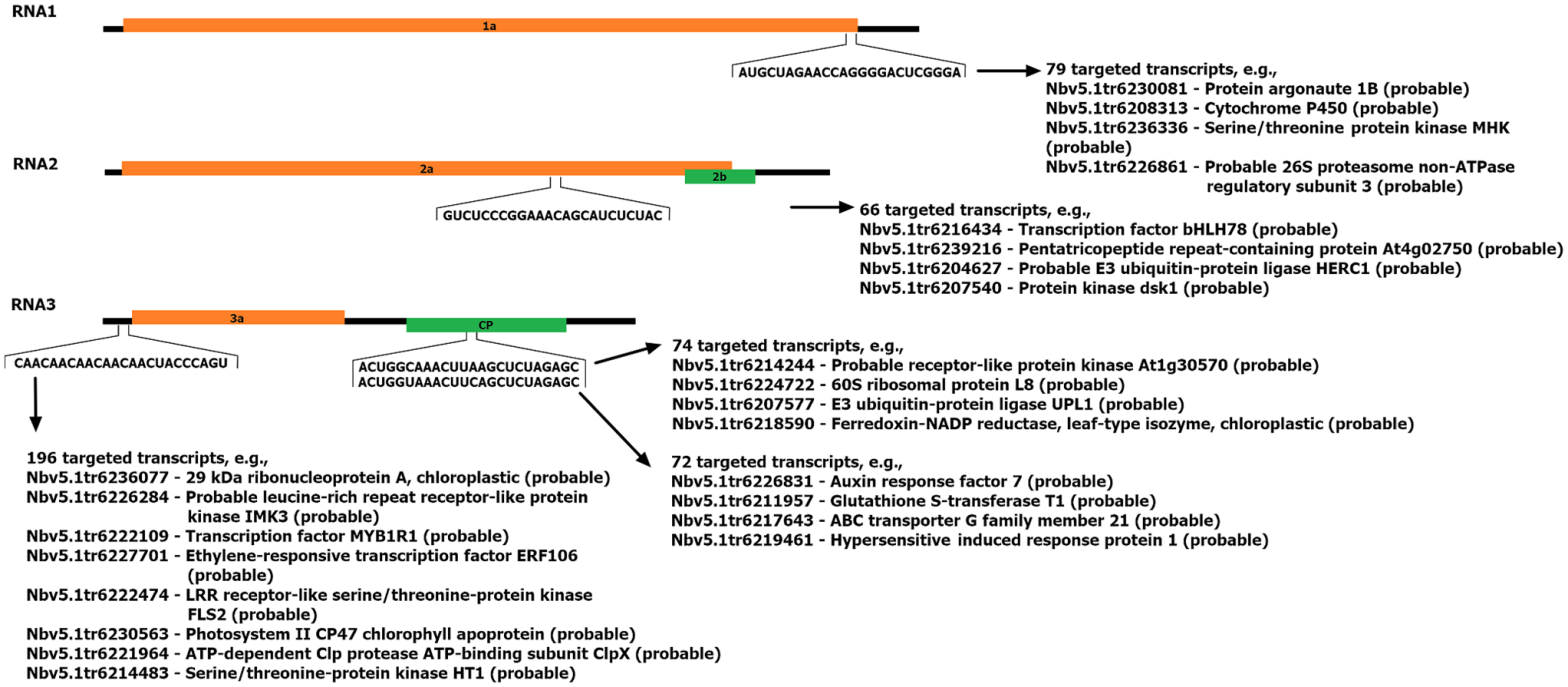
Schematic representation of sRNAs targeting PSV-P positions within viral genomic RNAs. The numbers of targeted potentially transcripts are shown with examples. The transcript accession numbers refer to transcriptome assembly v5.1 (Nakasugi et al. 2014)

miRNAs and ta-siRNA targets were searched among transcripts in the *N. benthamiana* transcriptome (Nbv5.1_transcriptome_primary_correct.fa). A total of 718 target transcripts were found for the identified miRNAs (Table S3C), and 3016 target transcripts were found for the ta-siRNAs (Table S3D). Moreover, complementarity between the miRNAs and the PSV and satRNA sequences was searched. miRNA and ta-siRNA targets were investigated among three different PSV strains and their satRNAs. No miRNA or ta-siRNA appeared to target these sequences. Therefore, whether sRNAs from outside the miRNA and ta-siRNA sets could target them was investigated. The search included all sRNAs that were sequenced at least ten times in at least one sample. At least one sequence fragment for each genomic RNA of all PSV strains was detected; however, no satRNA-directed sRNAs were identified (Fig. 1, Table S3E). Additionally, these PSV-specific sRNAs also were searched for targeting of *N. benthamiana* transcripts; the search revealed 664 target transcripts (Fig. 1, Table S3F).

**Fig. 2:**
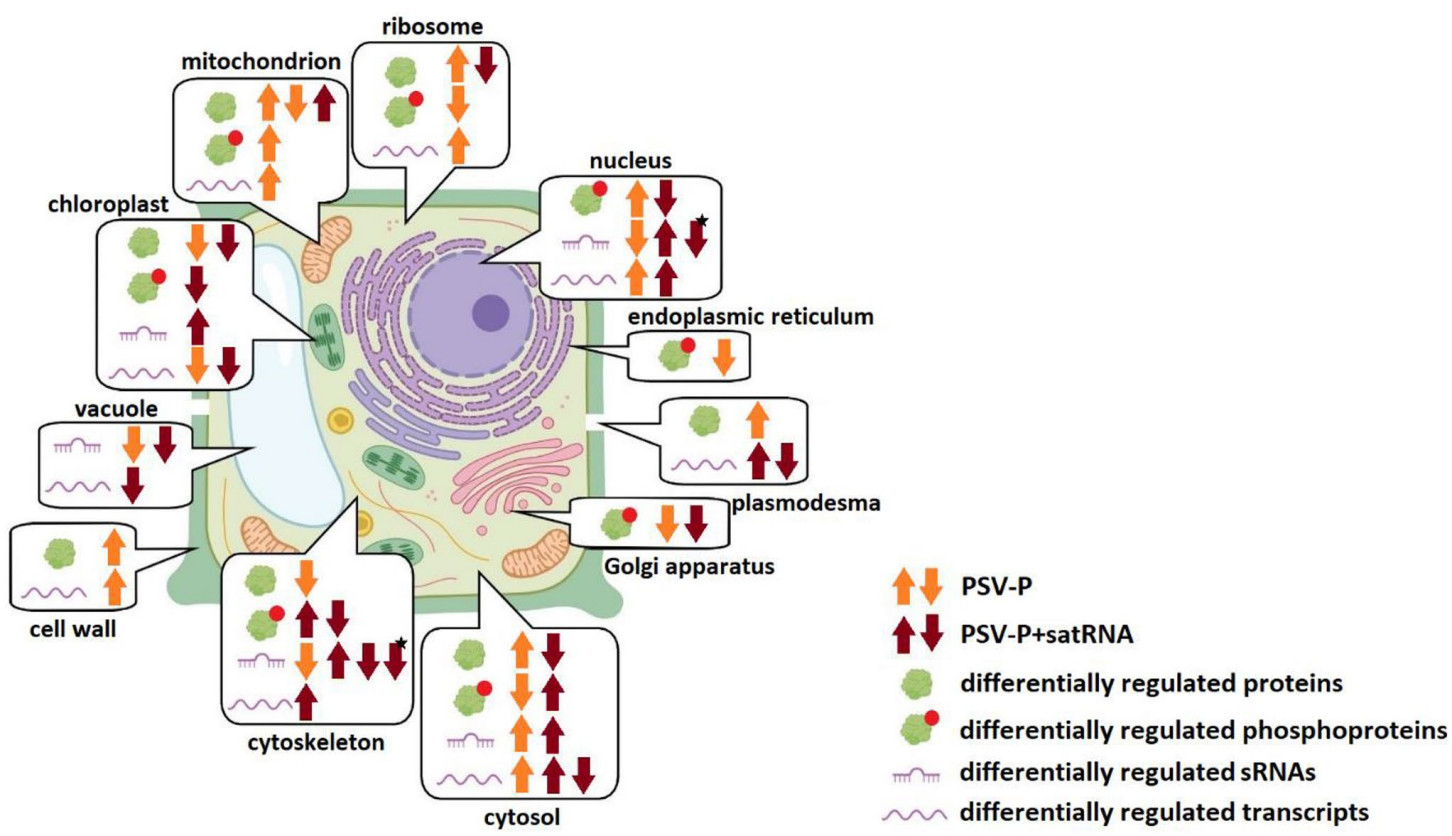
Graphical presentation of the results obtained in this study. Up and down arrows indicate up- or downregulated proteins, phosphoproteins, sRNAs, or transcripts. Orange and brown colored arrows are connected with the treatment of *N. benthamiana* plants with PSV-P and PSV-P + satRNA, respectively. Arrows are put next to the icons representing differentially regulated proteins, phosphoproteins, sRNAs targeting *N. benthamiana* transcripts, and transcripts associated with certain cellular components indicating their up- or downregulation. Black asterisk is associated with differentially regulated ta-siRNAs. The figure has been partially created using BioRender. A detailed list of the affected cellular components elements is presented in Table 2

The *N. benthamiana* transcriptome was used for transcript annotation to assign GO terms and perform enrichment analysis. Based on the results, transcripts targeted by identified miRNAs and ta-siRNAs for each condition were assigned to appropriate GO terms. With regard to cellular components, the upregulated miRNAs in PSV-P-treated plants were mainly associated with the cytosol, whereas the downregulated miRNAs were associated with the nucleus, cytoskeleton (Fig. 2, Table 2). In PSV-P + satRNA-infected plants, the upregulated miRNA-targeted transcripts were associated with the cytosol, nucleus, chloroplasts, and cytoskeleton, while the downregulated transcripts were associated with the cytoskeleton, lysosomes, and vacuoles. The downregulated ta-siRNAs in PSV-P + satRNA-infected plants targeted transcripts assigned to the nucleus and cytoskeleton.

**Table 2:**
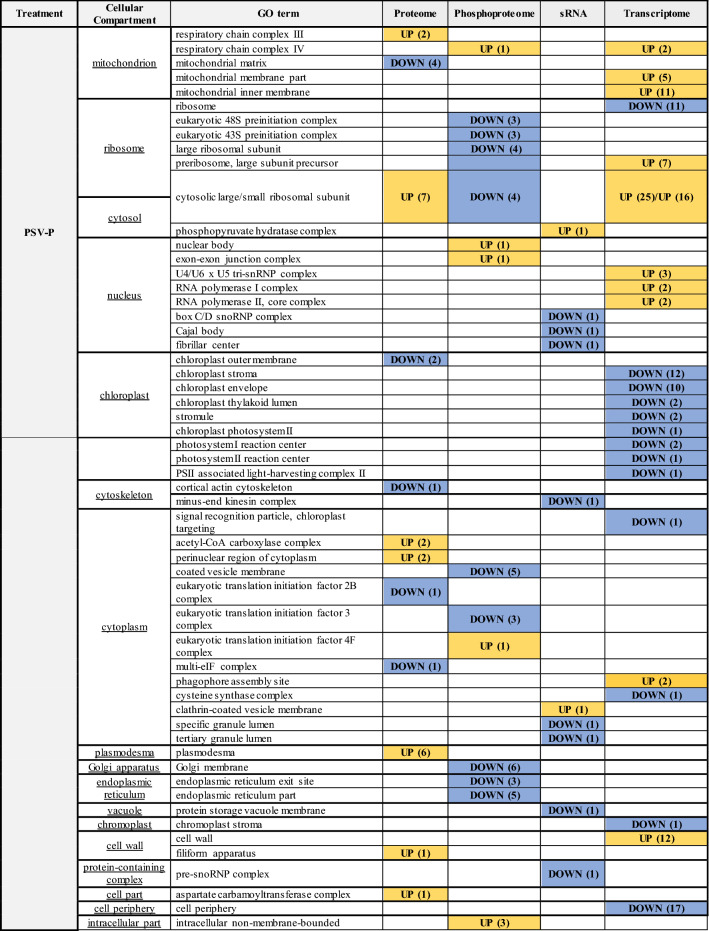

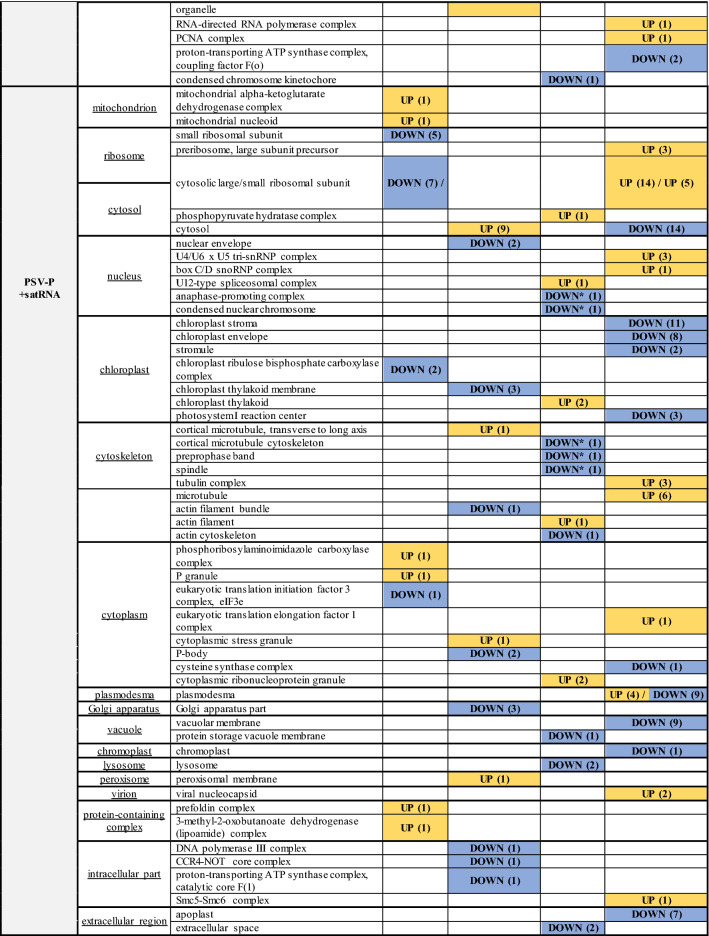
*Nicotiana benthamiana* cellular components (CC)affected by PSV-P and PSV-P + satRNA infection

UP/DOWN—up- or downregulation of proteins, proteins phosphorylation status, transcripts associated with given CC. In the case of sRNA analysis, up- or downregulation of miRNA or tasiRNA (*) targeted transcripts associated with given cellular component. The numbers in brackets represents the number of up- (yellow) or downregulated (blue) proteins, phosphoproteins, sRNAs targeted transcripts, and transcripts assigned to certain term

The biological processes affected by the presence of PSV-P and PSV-P + satRNA in *N. benthamiana* plants were analyzed (Table 3) and linked with the affected cellular components. Among the terms associated with the downregulated miRNAs in PSV-P-treated plants were ‘anastral spindle assembly involved in male meiosis’ and ‘snoRNA localization’, which are associated with the cytoskeleton and nucleus, respectively, while those in PSV-P + satRNA-treated plants included ‘actin filament depolymerization’, which is associated with the cytoskeleton, and ‘immune response’, which is associated with lysosomes. Several terms associated with the cytoskeleton and nucleus were noted among the terms connected with downregulated ta-siRNAs targeting transcripts (Table 3). These terms were ‘cortical cytoskeleton organization’, ‘preprophase band assembly’ and ‘microtubule cytoskeleton organization’ for the cytoskeleton and ‘ubiquitin-dependent protein catabolic process’, ‘regulation of circadian rhythm’, ‘defense response to bacterium, incompatible interaction’, ‘jasmonic acid mediated signaling pathway’, and ‘protein ubiquitination’ for the nucleus.

**Table 3:**
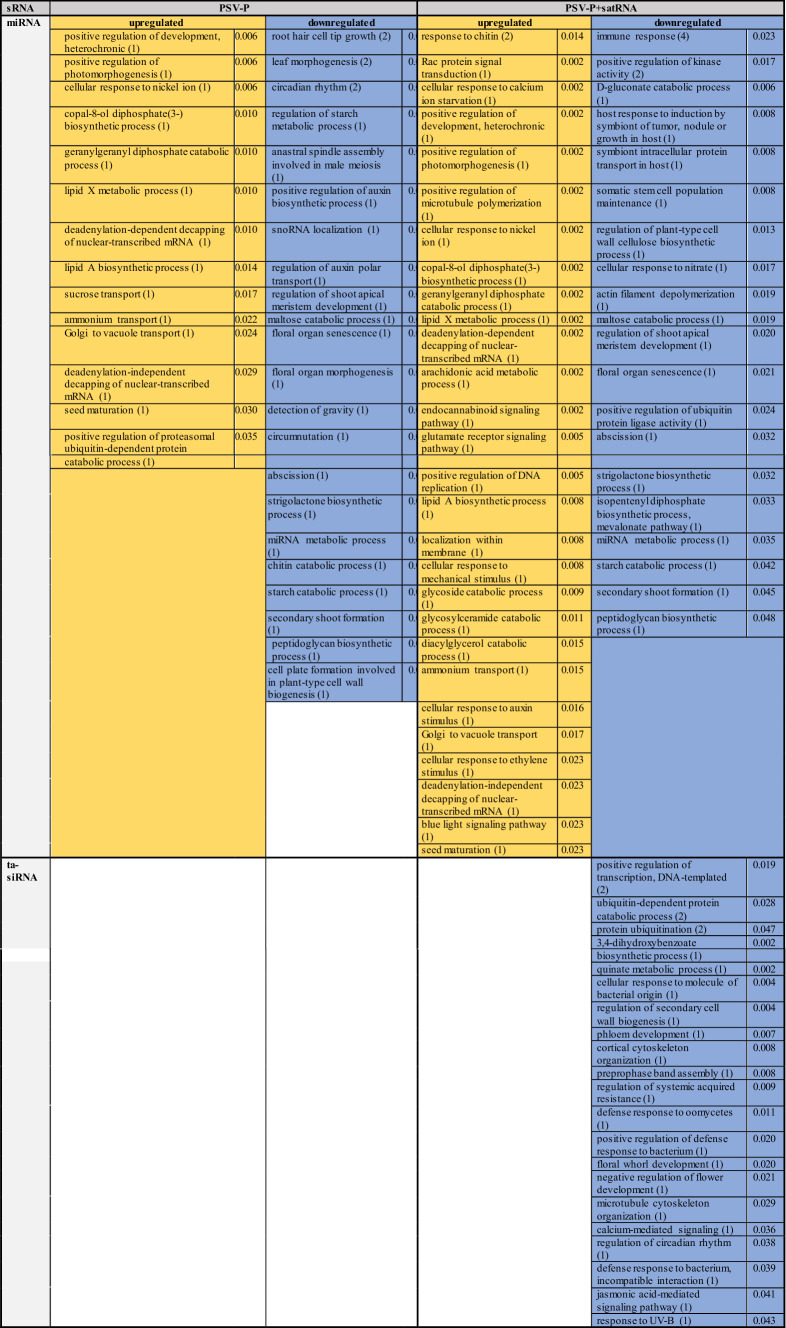
Enrichment analysis of *N. benthamiana* biological processes associated with differentially expressed sRNAs in PSV-P- and PSV-P + satRNA-infected plants

The numbers in parentheses are the numbers of up- (yellow) or downregulated (blue) sRNA-targeted transcripts assigned to the different terms. The values in the neighboring column are the *p* values from enrichment analysis

Moreover, in PSV-P- and PSV-P + satRNA-treated plants, several terms not related to given cellular components were related to developmental processes (e.g., ‘positive regulation of development’, ‘positive regulation of photomorphogenesis’, ‘regulation of shoot apical meristem development’, ‘phloem development’), carbohydrate metabolism (e.g., ‘regulation of starch metabolic process’, ‘maltose catabolic process’, ‘glycoside catabolic process’), nucleic acid metabolic processes (‘deadenylation-dependent decapping of nuclear-transcribed mRNA’, ‘miRNA metabolic process’, ‘positive regulation of DNA replication’, ‘positive regulation of transcription’) and responses to various stimuli such as hormones, inorganic substances or other organisms (e.g., ‘regulation of systemic acquired resistance’, ‘cellular response to molecule of bacterial origin’).

**Fig. 3:**
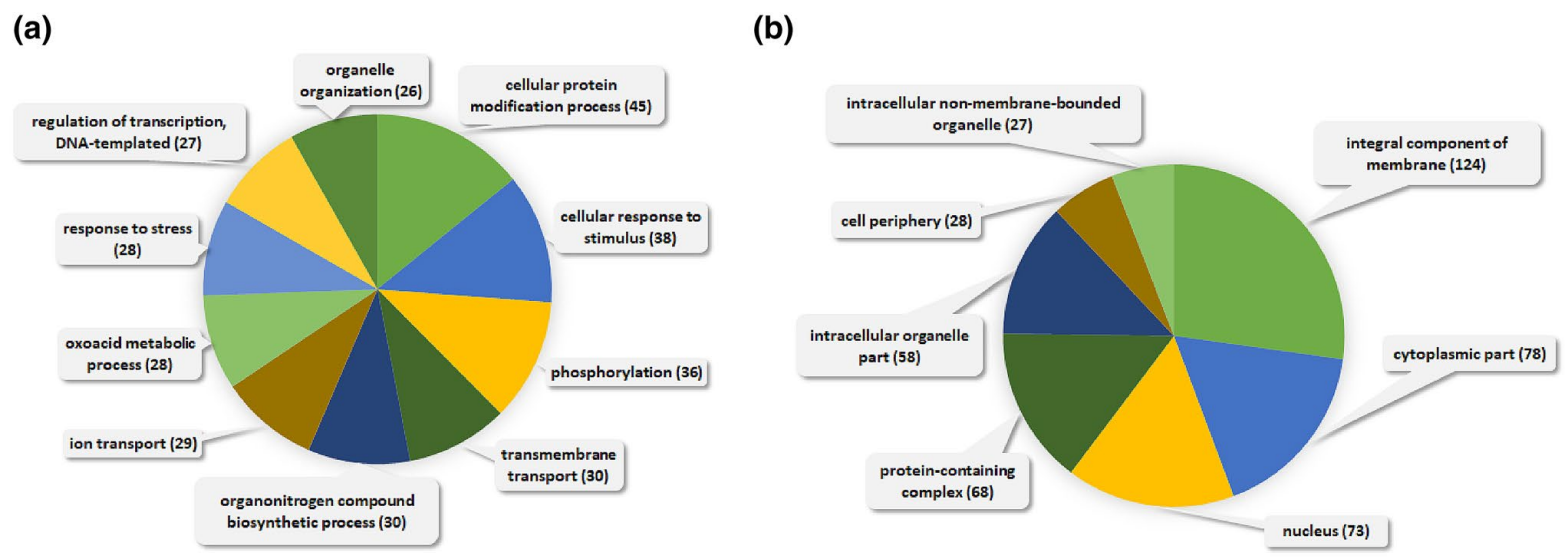
Biological process (**a**) and cellular component (**b**) terms for *N. benthamiana* PSV-specific sRNAs presented in pie charts**.** The numbers in parentheses represent the numbers of sRNA-targeted transcripts assigned to the different terms

GO analysis was performed for PSV-specific sRNAs that could also target *N. benthamiana* transcripts. The highest numbers of the targeted transcripts were associated with biological processes such as ‘cellular modification process’ (45), ‘cellular response to stimulus’ (38), and ‘phosphorylation’ (36) (Fig. 3a). Twelve kinase transcripts were detected as PSV-specific sRNAs (Table 4). With regard to cellular components many targeted transcript expression products were associated with the ‘integral component of membrane’ (124), ‘cytoplasmic part’ (78), and ‘nucleus’ (73) terms (Fig. 3b).

**Table 4:** A list of kinase genes detected as PSV-specific sRNA targets

*N. benthamiana* transcriptome ver. 5.1 accession number	GenBank accession number of the most similar sequence	UniProt accession number of the most similar sequence	Transcript name
Nbv5.1tr6226285	XM_019389806.1	A0A1J6IDC2	Probable leucine-rich repeat receptor-like protein kinase IMK3 (probable)
Nbv5.1tr6234589	XM_019391456.1	A0A1J6J380	G-type lectin S-receptor-like serine/threonine protein kinase At1g11330 (GsSRK) (probable)
Nbv5.1tr6230246	XM_019387400.1	A0A1J6JAM6	Probable adenylate kinase 2, chloroplastic (probable)
Nbv5.1tr6207540	XM_019370372.1	A0A1S3ZY14	Protein kinase dsk1 (probable)
Nbv5.1tr6229790	XM_016599785.1	A0A1J6HXJ5	L-type lectin-domain containing receptor kinase VIII.1 (probable)
Nbv5.1tr6219967	XM_009767702.1	A0A1S4B2E8	Ketohexokinase (probable)
Nbv5.1tr6225729	XM_009763275.1	A0A1U7Y2B6	Cysteine-rich receptor-like protein kinase 42 (probable)
Nbv5.1tr6214244	XM_016641048.1	A0A1U7VY33	Probable receptor-like protein kinase At1g30570 (probable)
Nbv5.1tr6222474	XM_016622510.1	A0A1S4AMV7	LRR receptor-like serine/threonine protein kinase FLS2 (probable)
Nbv5.1tr6214483	XM_009802777.1	A0A314KH12	Serine/threonine protein kinase HT1 (probable)
Nbv5.1tr6202246	XM_019372714.1	A0A1S4A707	Probable receptor-like protein kinase At1g33260 (probable)
Nbv5.1tr6206804	XM_009778236.1	A0A314L174	Bifunctional aspartokinase/homoserine dehydrogenase, chloroplastic (probable)
Nbv5.1tr6246208	XM_019389513.1	A0A1S4CG10	Probable receptor-like protein kinase At5g15080 (similar to)
Nbv5.1tr6226274	XM_009801385.1	A0A1U7Y6D2	Mitogen-activated protein kinase 9 (MAPK9) (probable)
Nbv5.1tr6225580	XM_019368453.1	A4USB3	4-diphosphocytidyl-2-*C*-methyl-d-erythritol kinase, chloroplastic/chromoplastic (probable)
Nbv5.1tr6236314	XM_009759479.1	A0A314KZN1	1-phosphatidylinositol 3-phosphate 5-kinase FAB1 (probable)
Nbv5.1tr6236336	XM_016577129.1	A0A1U7XLG1	Serine/threonine protein kinase MHK (probable)
Nbv5.1tr6234411	XM_009769672.1	A0A1U7W004	Probable serine/threonine protein kinase At1g18390 (probable)
Nbv5.1tr6228893	XM_016641701.1	A0A1S4C7F6	Cysteine-rich receptor-like protein kinase 10 (probable)
Nbv5.1tr6219249	XM_016613652.1	A0A1J6IHZ1	Probable LRR receptor-like serine/threonine protein kinase At5g48740 (probable)
Nbv5.1tr6230398	XM_019378300.1	A0A1U7X4A5	Leucine-rich repeat receptor-like protein CLAVATA2 (probable)
Nbv5.1tr6207031	XM_009799211.1	A0A1U7Y304	Uncharacterized protein sll0005 (probable)
Nbv5.1tr6205665	XM_009790524.1	A0A1U7XGP2	Uncharacterized WD repeat-containing protein alr3466 (probable)

sRNA-seq and phosphoproteomic data associations were searched in the context of cellular components. Changes in protein phosphorylation level and levels of sRNAs targeting *N. benthamiana* transcripts were found in the nucleus and cytosol in PSV-P-infected plants, whereas, in PSV-P + satRNA-treated plants—chloroplast, nucleus, cytosol, and cytoskeleton (Table 2). However, proteins with altered phosphorylation levels were not among the targets of the identified sRNAs in this study.

### Transcriptomic analyses of *N. benthamiana* infected with PSV-P (and satRNA) indicate numerous changes in transcripts associated with ribosomes, the cytosol, and chloroplasts

**Fig. 4:**
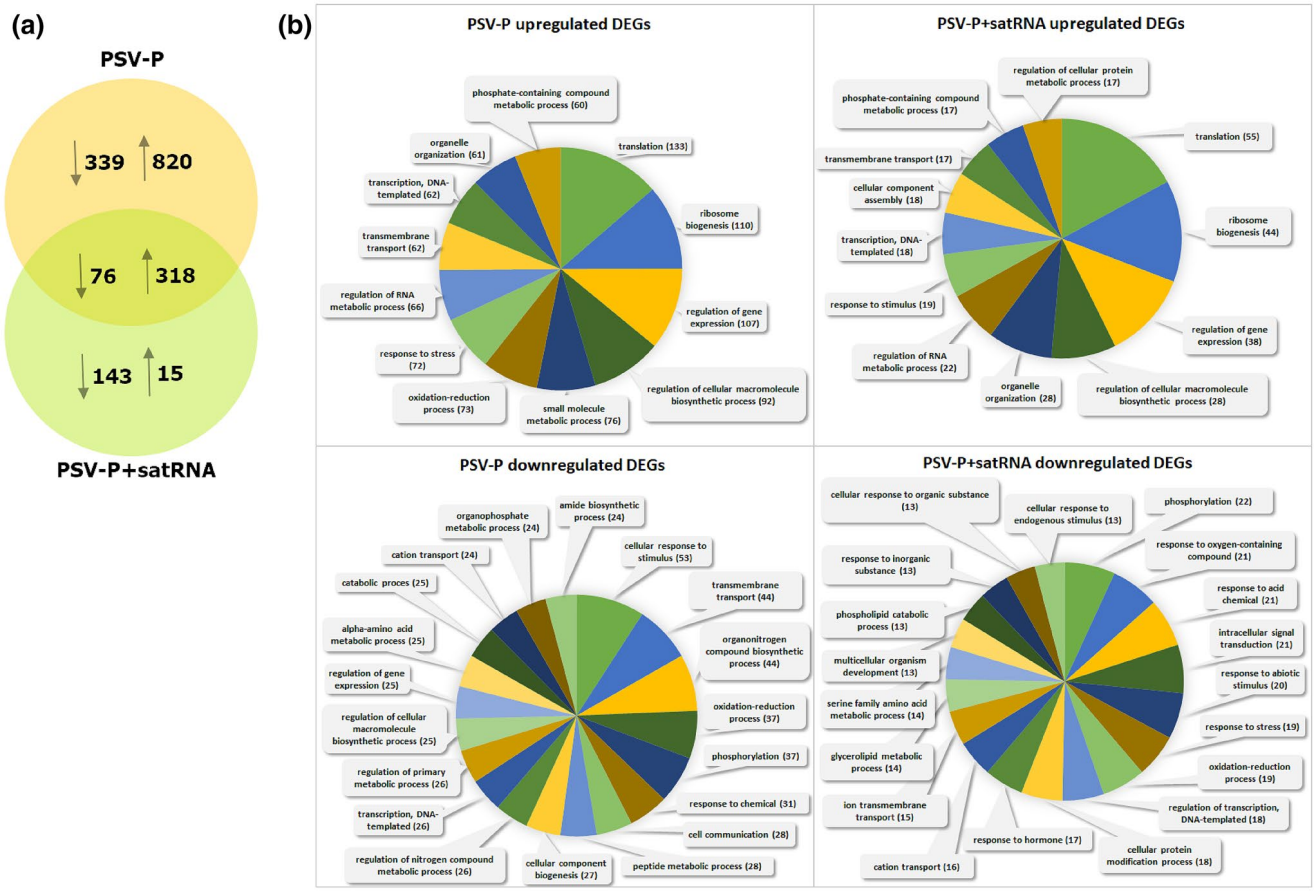
**a** Venn diagram showing the unique and common differentially expressed genes (DEGs) in PSV-P and PSV-P + satRNA plants. The arrows indicate upregulated (↑) and downregulated (↓) DEGs. **b** Gene Ontology biological process terms for the DEGs of *N. benthamiana* infected with PSV-P or PSV-P + satRNA presented in pie charts. The distribution of GO terms was analyzed separately for the upregulated and downregulated DEGs. The specific cellular component terms are presented in Table 2

Species-specific microarrays (Nb-105 k) (Goralski et al. 2016) were used to evaluate *N. benthamiana* plant responses to PSV-P or PSV-P + satRNA infection at the transcriptomic level. Transcriptome profiling revealed 14,138 DEGs (Table S4A); 1553 DEGs were identified in PSV-P-infected *N. benthamiana* compared with noninfected *N. benthamiana* (*p* value < 0.05, at least a twofold change) (Table S4B) while 552 DEGs were identified in PSV-P + satRNA-infected plants compared to noninfected plants (*p* value < 0.05, at least a twofold change) (Table S4C). In both experimental conditions, the number of upregulated DEGs was higher than the number of downregulated DEGs; however, the difference between the number of up- and downregulated DEGs was larger in the PSV-P-infected plants than in the PSV-P + satRNA-treated *N. benthamiana* (Fig. 4a).

GO enrichment analysis of the cellular component terms of the downregulated DEGs in PSV-P-infected *N. benthamiana* revealed that the most affected cellular components were chloroplasts and ribosomes, whereas in PSV-P + satRNA-infected plants, the most affected cellular components were chloroplasts and the cytosol (Fig. 2, Table 2). The upregulated DEGs in PSV-P-infected plants were mostly associated with mitochondria, ribosomes, the cytosol, cell wall, and the nucleus, while those in PSV-P + satRNA-infected plants were mostly associated with ribosomes, PD, vacuole, and the cytoskeleton.

The biological processes affected by the presence of PSV-P and PSV-P + satRNA (Table 5) and their associations with cellular components were determined. Among the identified terms associated with downregulated transcripts in PSV-P-treated plants were ‘chlorophyll biosynthetic process’, ‘photosynthesis, dark reaction’, and ‘protein import into chloroplast thylakoid membrane’, which are associated with chloroplasts, and ‘translation’, which is associated with ribosomes. In PSV-P + satRNA-treated plants, the terms associated with the downregulated transcripts that were related to chloroplasts included ‘protein-chromophore linkage’ and ‘photosynthesis, light harvesting’; those related to the cytosol included ‘serine family amino acid biosynthetic process’; and those related to responses to stresses included ‘cold acclimatization’, ‘cellular oxidant detoxification’ and ‘response to oxidative stress’. Among the annotated terms for the upregulated transcripts in PSV-P-treated *N. benthamiana*, several terms were associated with ribosomes, e.g., ‘cytoplasmic translation’, ‘regulation of translational elongation’ (both of which are also associated with the cytosol), ‘ribosomal small subunit assembly’, and ‘endonucleolytic cleavage’; with the nucleus, e.g., ‘transcription by RNA polymerase I’, transcription, or maturation of LSU-rRNA; and with mitochondria, e.g., mitochondrial electron transport from NADH to ubiquinone and from cytochrome c to oxygen. In PSV-P + satRNA-treated plants, the terms annotated to upregulated transcripts that were associated with ribosomes were ‘cytoplasmic translation’, ‘regulation of translational elongation’, and ‘maturation of SSU-rRNA’; those associated with the cytoskeleton were ‘microtubule-based process’, ‘cellular response to gravity’, ‘endosperm cellularization’, ‘regulation of root morphogenesis’, and ‘phragmoplast assembly’; and those associated with the nucleus included ‘mRNA splicing, via spliceosome’.

**Table 5:**
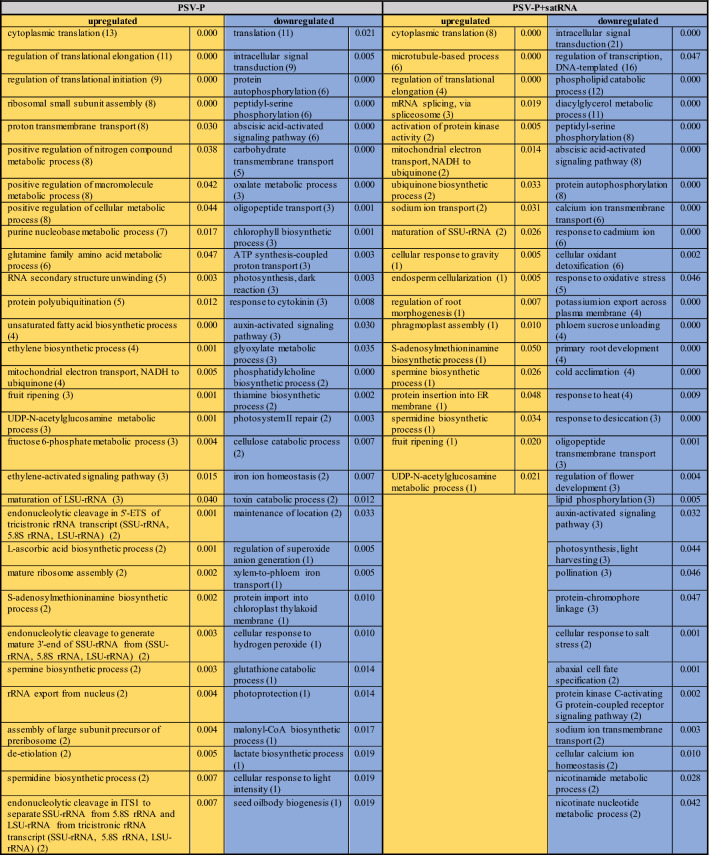

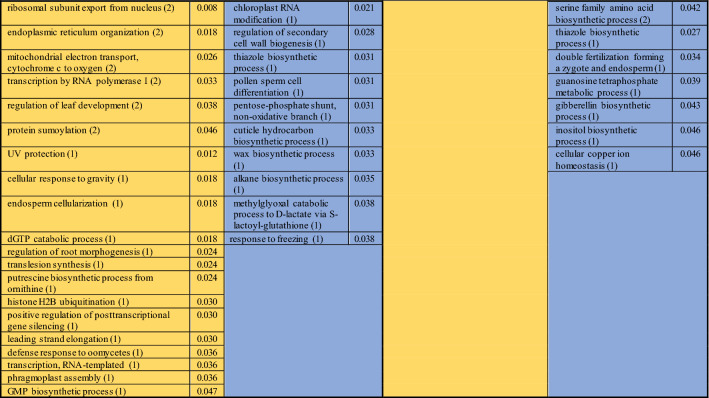
Enrichment analysis of *N. benthamiana* biological processes associated with the differentially regulated transcripts in PSV-P- and PSV-P + satRNA-infected plants

The numbers in parentheses are the numbers of up- (yellow) or downregulated (blue) transcripts assigned to the different terms. The values in the neighboring column are the *p* values from enrichment analysis

Terms associated with signal transduction, such as ‘intracellular signal transduction’, ‘abscisic acid-activated signaling pathway’, ‘auxin-activated signaling pathway, and ‘protein kinase C-activating G protein-coupled receptor signaling pathway’, were mostly downregulated in both conditions; however, more terms were reported for PSV-P + satRNA-treated *N. benthamiana* than for PSV-P-treated *N. benthamiana*. A similar situation was observed with regard to phosphorylation, which is a regulator for some signal transduction pathways. Nonetheless, the upregulation of transcripts connected with ‘activation of protein kinase activity’ was also observed.

In general, translation (133 and 110), ribosome biogenesis (110 and 44), and regulation of gene expression (107 and 38) were the most upregulated processes (the numbers in parentheses indicate the identified transcripts in PSV-P- and PSV-P + satRNA-infected *N. benthamiana,* respectively) (Fig. 4b). Among the terms associated with downregulated transcripts, ‘phosphorylation’ was significantly altered. There were more DEGs in PSV-P-treated plants than in PSV-P + satRNA-treated plants.

### Proteomic and phosphoproteomic analyses of *N. benthamiana* infected with PSV-P (and satRNA) indicate numerous changes in (phospho) proteins associated with ribosomes, the cytosol, mitochondria, and chloroplasts

To gain insight into the cellular compartments affected by the presence of PSV-P and PSV-P + satRNA, GO enrichment analysis was performed on a previously obtained dataset (Wrzesińska et al. 2018). Numerous cellular compartments were influenced by PSV-P and PSV-P + satRNA infection (Fig. 2, Table 2). In PSV-P-treated plants proteins associated with mitochondrial parts were both upregulated and downregulated. Ribosome-, cytosol-, and PD-associated proteins level was upregulated, while chloroplast and cytoskeleton proteins were less abundant. Similarly, in PSV-P + satRNA-treated plants mitochondrion- and chloroplast-associated proteins level was upregulated and downregulated, respectively. However, contrary to PSV-P-treated *N. benthamiana*, decrease in the abundance of ribosome- and cytosol-associated proteins was observed (Fig. 2, Table 2).

With regard to the protein phosphorylation levels, in PSV-P-treated plants mitochondrial phosphoproteins were more abundant, while these associated with the ribosome and ER were decreased. The cytoplasmic phosphoproteins were both increased and decreased in abundance, while Golgi apparatus—decreased, in plants infected with PSV-P as well as PSV-P + satRNA. In the nucleus, phosphoproteins displayed altered abundance (increase and decrease in PSV-P and PSV-P + satRNA plants, respectively). Moreover, chloroplast and cytosolic phosphoproteins were increased and decreased in abundance, respectively. The levels of the phosphorylated proteins associated with the cytoskeleton were more and less abundant in PSV-P + satRNA-infected *N. benthamiana* plants (Fig. 2, Table 2).

### Protein–protein interaction network analysis pinpoints an involvement of miRNA regulation in glycolysis/gluconeogenesis, oxidative phosphorylation, and nitrogen metabolism

To integrate the resulting data from ‘omics’ analyses, STRING database was utilized to present the protein–protein interaction networks associated with certain differentially regulated pathways. Of the pathways regulated by differentially expressed miRNAs in PSV-treated *N. benthamiana* were glycolysis/gluconeogenesis, fatty acid degradation, and tyrosine metabolism (Fig. S2A). These pathways include alcohol dehydrogenase 1 (Niben101Scf04078g01006.1), which is the potential target of miRNA exhibiting similarity to miR169 (which level was downregulated). Another, miR169 potential target—a sequence of ATP synthase subunit alpha (Niben101Scf00167g04011.1) was shown to interact with proteins associated with oxidative phosphorylation. These interacting proteins were shown to be upregulated both, in transcriptomic and proteomic analyses. Moreover, another miRNA similar to miR319, which level was upregulated, potentially targets the sequence of enolase (Niben101Scf06769g04014.1), interacts with a group of proteins associated with glycolysis/gluconeogenesis. MiRNA target transcripts, however, have not been found among differentially regulated proteins or transcripts, which could be influenced by the time point in which analysis was performed. At this time, miRNA effect on all the target transcripts could not be detected yet. Additionally, transcripts or proteins associated with specific cellular components were marked. Transcripts connected with oxidative phosphorylation and starch and sucrose metabolism were associated with cytoplasm, chloroplast, and mitochondrion.

In PSV-P + satRNA-treated plants, an involvement of downregulated miRNA similar to miR157, which targets were shown to participate in nitrogen metabolism and terpenoid backbone biosynthesis, namely high affinity nitrate transporter 2.5 (Niben101Scf12671g00007.1), carbonic anhydrase (Niben101Scf02502g07009.1), hydroxymethylglutaryl-CoA synthase (Niben101Scf01111g01003.1) (Fig. S2B). Similarly to PSV-P-infected plants, miR319 was also shown to regulate the glycolysis/gluconeogenesis process. Here, transcripts or proteins associated with specific cellular components were found, however, just one transcript was connected with cytoplasm and intracellular membrane bounded organelle.

Moreover, miRNA target transcripts were searched among transcriptomic results (Table 1B). For downregulated miR169 for both PSV-P and PSV-P + satRNA-infected plants, upregulated protein argonaute 1B (AGO1B) and nuclear transcription factor Y subunit A-6 genes were found. Potential targets of the downregulated miR6148 were the upregulated tubulin alpha chain and 60S ribosomal proteins in PSV-P infected *N benthamiana*. Moreover, miR157 in PSV-P + satRNA-treated plants displayed decreased levels, and its target—squamosa promoter-binding-like protein 9 resulted in the upregulation. The potential target of the upregulated miR4376—DAG protein was slightly downregulated, while the potential target of downregulated ta-siRNA—DNA polymerase theta displayed a slightly increased level in PSV-P + satRNA-infected plants.

### Data validation

For the validation of microarray transcriptomic data, six DEGs, upregulated in PSV-P infected plants were chosen: *MBF1C*, *PR1*, *EF1delta*, *AGO2*, *PNO1*, and *PR2*. Except for *PR1* and *PR2*, these genes were also identified as upregulated DEGs in PSV-P + satRNA infected plants. RT-qPCR analysis confirmed the direction of expression changes for all of them (Table S5), although in the case of *EF1delta* and *PNO1*, the increase in transcript level was lower than twofold. Additionally, four DEGs, downregulated in PSV-P-infected plants were chosen: *PPCK*, *IRT1*, *PAP1*, and *ABCC*. The first two of them were also identified as downregulated DEGs in PSV-P + satRNA infected plants. RT-qPCR analysis confirmed the direction of expression changes of these genes, except for *IRT1* in plants co-infected with satRNA, where a slight (~ 20%) increase in transcript level was observed. Altogether, RT-qPCR results were concordant with the results based on microarray gene expression analysis.

The expression level of four chosen kinases genes detected as PSV-specific sRNA targets were checked at 5 dpi (Table S6). In PSV-P-treated plants, the levels of all analyzed kinases genes expression were down-regulated. In PSV-P + satRNA-treated plants, the expression of three kinases genes was down-regulated and the expression of one gene was up-regulated, however, the results for *GsSRK* and *FLS2* genes were not statistically significant. Our previous phosphoproteomic analyses indicated strong down-regulation of the phosphorylation level in PSV-P-treated *N. benthamiana* plants, which was consistent with RT-qPCR analyses, where the majority of the analyzed genes were down-regulated.

To validate miRNA expression levels, stem-loop RT-qPCR was implemented. Two miRNAs, which levels were changed both in PSV-P and PSV-P + satRNA plants, were chosen for verification. MiR169 and miR319 expression levels measured by RT-qPCR were in agreement with sRNA-seq results, however, miR319 expression value in PSV-P + satRNA-treated plants was not statistically significant (Table S7). Additionally, the change in the expression of miR169 target gene—*AGO1B* was verified. AGO1B transcript level was upregulated in our previous studies (Obrępalska-Stęplowska et al. 2018) and according to transcriptomic analysis in this study, *AGO1B* expression level was upregulated 2.491 fold in PSV-P-infected plants and 2.180 fold in plants co-infected with PSV-P and satRNA (with *p* values 0.002 and 0.017, respectively) (Table 1). RT-qPCR analysis in plants at 5 dpi also revealed a slight increase in its expression level (1.486 fold with *p* value 0.000 in PSV-P-infected plants and 1.303 fold with *p* value 0.0030 in PSV-P + satRNA-infected *N. benthamiana*).

The changes in the expression of miRNAs, their targets, and kinases are not high. The reason might have been the shift in pathogenesis progress in the harvested material for sRNA-seq analyses and for the validation although the samples for validation were collected at the same days post inoculation as ‘omics’ analyses. Plants were grown during different seasons, which may have influenced the infection dynamics. The changes in miRNA expression levels were not high, therefore, the changes in their target expression level were not expected to be high. Moreover, the discrepancies in miRNA validation (lower expression level resulting from RT-qPCR analysis compared to RNA-seq analysis) in *N. benthamiana* plants were also observed in other studies (Baksa et al. 2015).

## Discussion

High-throughput approaches enable global examination of various processes affected in response to exposure to biotic and abiotic factors. The aim of our studies was to analyze sRNAs, transcripts, proteins, and phosphoproteins affected during PSV-P–*N. benthamiana* interaction in the context of their cellular localization or functional connections with particular cellular compartments. Our PSV-P–satRNA–*N. benthamiana* pathosystem data are based on previously published (phospho)proteomic data (Wrzesińska et al. 2018) and on new sRNA and transcriptomic data (this manuscript) on plant virus–satRNA–host interactions.

Our sRNA-seq analysis revealed that the majority of the sequenced sRNAs belonged to the 24 nt size class followed by 20 nt size class. Usually, 21 nt, 22 nt, and 24 nt size classes of sRNA are yielded, however, the high level of 20 nt sRNA has been observed in few studies as well (Qu et al. 2008; Xu et al. 2012). The mechanism of the biogenesis and function of 20 nt sRNA are still elusive, however, it has been reported that 20 nt sRNA size class likely result from partial degradation of the 21 nt sRNAs (Herranz et al. 2015).

The chloroplast is known to be very important during plant–virus interactions (Zhao et al. 2016). Its membranes are exploited by replicating viruses (Budziszewska and Obrępalska-Stęplowska 2018). In this study, we found that this organelle was one of the cell compartments most affected by PSV infection. PSV-P infection without satRNA resulted in a higher number of downregulated chloroplast- and photosynthesis-associated transcripts (38) than PSV-P infection with satRNA (22), such as chlorophyll a/b-binding proteins and photosystem I and II proteins, which have been found to be downregulated in different plant–virus interaction-related studies (Bhattacharyya et al. 2015; Mochizuki et al. 2014; Pineda et al. 2010). Mosaic or chlorosis symptoms in virus-infected leaves are caused by disruption of normal chloroplast function (Cheng et al. 2008; Xu and Nagy 2010), which is likely associated with changes in the levels of proteins and protein phosphorylation, sRNAs, and transcripts associated with the chloroplast stroma, thylakoids, envelope, photosystems, and light harvesting-complex, which are involved in virus-induced disease and immunity signaling (Bhat et al. 2013; Kangasjärvi et al. 2014; Zhao et al. 2016). The protein levels of the ribulose bisphosphate carboxylase large and small chains as well as ribulose bisphosphate carboxylase/oxygenase (RuBisCO) activase were downregulated in PSV-P + satRNA-treated plants. Of note, RuBisCO interacts with potato virus Y (PVY) CP, and this interaction may be involved in the production of mosaic and chlorosis symptoms (Feki et al. 2005). Moreover, the transcript level of photosystem II CP47 chlorophyll apoprotein (psbB), targeted by PSV-specific sRNAs, was downregulated based on our transcriptomic data. Significant decreases in the abundance of psaA and psbB in photosystems I and II have been reported in TMV-infected *N. tabacum* (Das et al. 2019).

Mitochondria, which are responsible for respiration, have also been reported to play an important role in host defense against pathogens such as viruses. (Lam et al. 2001; Nie et al. 2015; Tognetti et al. 2012; Welchen et al. 2014). Mitochondrial alpha-ketoglutarate dehydrogenase subunit E2 (kGDH E2) that was upregulated in PSV-P + satRNA–infected plants at the proteomic level in our study, has been found to function as a salicylic acid (SA)-binding protein in tomato. It has been demonstrated that the binding of SA by kGDH E2 is upstream of and affects the activity of the miETC and plays an important role in basal defense against tobacco mosaic virus (TMV). SA does not enhance TMV defense in kGDH E2-silenced tomato plants but does reduce TMV susceptibility in *N. benthamiana* plants transiently overexpressing kGDH E2 (Liao et al. 2015).

Other cell compartments associated with the altered sRNA, transcripts, proteins, and phosphorylation statuses were the cytosol and ribosomes. Plant virus components are translated by the host’s cellular machinery, including ribosomes, in the cytoplasm. Interestingly, in our study, at the early stage of infection, proteins associated with ribosomes and the cytosol were upregulated in PSV-P-infected plants and downregulated in PSV-P + satRNA-treated plants; however, the numbers of downregulated transcripts in the latter condition at the later stage of infection were lower than those of the upregulated transcripts, which suggests that satRNA at the beginning of infection causes reprogramming of the host’s synthesis machinery and response reaction (Obrępalska-Stęplowska et al. 2018). A comparison of our ribosome-associated data obtained through different ‘omics’ analyses revealed that in PSV-P-treated plants ribosome-associated proteins and transcripts were upregulated; in contrast, in PSV-P + satRNA-treated plants, those proteins were downregulated at the early stage of infection, while the corresponding transcripts were upregulated after symptom development. Host ribosomal proteins are essential for viral RNA translation; therefore, an increase in these proteins has been associated with virus infection (Yang et al. 2009). Additionally, satRNA influenced the pathogenesis process, changes in host proteins and transcripts, and caused differences in virus accumulation levels. Our data also confirmed previous data on changes in the ribosomal and cytosolic levels of proteins caused by the presence of viruses (Amuge et al. 2017; Das et al. 2019; Stare et al. 2017).

Another cellular structure affected by the presence of plant viruses is the cytoskeleton. In our study, PSV-P infection downregulated cortical actin cytoskeleton proteins and minus-end kinesin complex-targeting miRNAs, while PSV-P + satRNA infection affected cortical microtubules by upregulating the phosphorylation levels of the associated proteins as well as upregulating microtubules at the proteomic level, which may have resulted from the downregulation of ta-siRNAs targeting cortical microtubule cytoskeleton transcripts. In several studies, TMV has been shown to form a VRC of genomic RNA, MPs, and replication-associated proteins at ER–plasma membrane contact sites. Such findings have implied that plant viruses actively employ microtubule cytoskeleton systems for virion and VRC movement from the replication site to PDs (Boyko et al. 2007; Liu and Nelson 2013). Moreover, it has been suggested that microtubules function to promote the proteasomal degradation of MPs (Gillespie et al. 2002; Reichel and Beachy 1998), while actin microfilaments have been found to be involved in plant virus movement (Harries et al. 2009; Kawakami et al. 2004). As mentioned above, microtubule-related terms were associated with upregulated molecules in PSV-P + satRNA-infected plants. Additionally, sRNA profiling revealed changes in protein ubiquitination and ubiquitin-dependent protein catabolic processes, which are associated with proteolysis.

Our study not only included cellular components analysis but also revealed additional data associated with kinases and their potential regulatory factors (on the sRNA, transcript, and phosphoprotein levels). Moreover, virus-specific sRNAs in the sRNA pool were identified to contribute to the regulation of numerous cellular functions, including those of transcripts/proteins associated with different organelles, RNA silencing, kinase activity, antiviral defense, and symptom development. Our previous phosphoproteomic data revealed strong downregulation of overall protein phosphorylation levels after infection (Wrzesińska et al. 2018); this finding was supported by our new sRNA and transcriptomic data, which indicated that PSV-specific sRNAs targeting *N. benthamiana* transcripts were substantially associated with phosphorylation. In our study, numerous PSV-specific sRNAs that target kinases, which are enzymes mediating protein phosphorylation (a major mechanism of signal transduction), were identified. One such is RLK proteins group, which is involved in plant–pathogen interactions and defense responses. For example, the leucine-rich repeat (LRR) receptor-like serine/threonine protein kinase FLS2, which activation in *Arabidopsis thaliana* was found to directly or indirectly be associated with activation of downstream signals and plant defense responses (Afzal et al. 2008; Chinchilla et al. 2006; Haffani et al. 2004). Silencing of such kinase genes may contribute to the disruption of signaling pathways regulated by phosphorylation, which is considered to be a major control mechanism for protein activity in plant–pathogen interactions (Friso and van Wijk 2015; Kersten et al. 2009). In this study, RT-qPCR expression analysis of the chosen kinases genes revealed a decrease in the levels of all their transcripts in both PSV-P- and PSV-P + satRNA-infected *N. benthamiana* except for *FLS2*, which level was downregulated in PSV-P-infected plants and insignificantly upregulated in the plants infected with the virus and satRNA. This result may be connected with our previous phosphoproteomic observations, where PSV-P infection resulted in the considerable decrease in the proteins phosphorylation level, while the addition of satRNA caused phosphorylation level increment (Wrzesińska et al. 2018). Plant virus-induced downregulation of phosphorylation has been recently confirmed by another phosphoproteomic study (Lu et al. 2019). In addition, in this study, transcript analysis revealed a higher number of downregulated transcripts connected with phosphorylation in PSV-P-infected plants (without satRNA) than in PSV-P + satRNA-infected plants; this finding is consistent with our transcriptomic analysis of another PSV strain, G, which revealed that satRNA addition accelerated and exacerbated symptoms (Obrępalska-Stęplowska et al. 2018). The enrichment of intracellular signal transduction and protein autophosphorylation among the downregulated transcripts in the PSV-P- and PSV-P + satRNA-infected plants in this study confirms the significance of these processes in plant–virus interactions. Additionally, in our study the changes in the level of protein phosphorylation playing a role in signal transduction as well as the changed level of PSV-specific sRNAs targeting *N. benthamiana* transcripts involved in phosphorylation/dephosphorylation processes were found, however, PSV-specific sRNAs targets associated with phosphorylation were not found among the proteins with altered phosphorylation level at the analyzed time point.

Apart from multiple kinases, several transcription factors were identified among the PSV-specific sRNA-targeted *N. benthamiana* transcripts, such as ethylene-responsive transcription factor (ERF), which have been reported to be involved in resistance against pathogens (Fischer and Dröge-Laser 2004). Interestingly, the protein argonaute 1B, a component of RISC, was also found to be a PSV-specific sRNA target in this study. Identification of targets of PSV-specific sRNAs involved in the host defense response against pathogens implies that RNA silencing by pathogen RNA-derived siRNAs may be a counterdefense mechanism against the host.

In conclusion, PSV-P and PSV-P + satRNA infection resulted in significant changes in several *N. benthamiana* cell compartments, including mitochondria, chloroplasts, ribosomes, the cytosol, the nucleus, and the cytoskeleton, at the proteome, phosphoproteome, transcriptome and sRNA-ome levels. Changes in mitochondria were found in PSV-P-infected plants in most ‘omic’ analyses, whereas changes in chloroplasts were found in PSV-P + satRNA-infected plants in most analyses. Virus presence affected important processes such as photosynthesis, translation, transcription, and mRNA splicing. Moreover, new transcriptomic and sRNA data associated with PSV-P and PSV-P + satRNA infection in *N. benthamiana* plants were obtained that will be applicable to a wide range of studies on cucumovirus and satRNA pathogenesis.

## Supplementary Information

Below is the link to the electronic supplementary material.

Supplementary file1 Figure S1. Size distribution of the redundant sRNA sequence reads. The analysis was performed on four replicates for each condition. (TIF 22 kb)

Supplementary file2 Figure S2. Networks of potential protein–protein interactions of PSV-P-responsive (A) and PSV-P+satRNA-responsive (B) proteins from N. benthamiana (retrieved from proteomic, transcriptomic, and sRNA-seq analyses), differentially changed in their abundance. (TIF 684 kb)

Supplementary file3 (DOCX 16 kb)

Supplementary file4 (DOCX 16 kb)

Supplementary file5 (XLSX 353 kb)

Supplementary file6 (XLSX 6253 kb)

Supplementary file7 (DOCX 17 kb)

Supplementary file8 (DOCX 16 kb)

Supplementary file9 (DOCX 16 kb)

## Data Availability

Small RNA sequencing data were deposited into the Gene Expression Omnibus with the dataset identifier GSE128200. Transcriptome data of the PSV-P- and PSV-P + satRNA-infected *N. benthamiana* were deposited in the Gene Expression Omnibus with the dataset identifier GSE133124.
