# Supplementary figures and images for: Multiple cellular compartments engagement in Nicotiana benthamiana-peanut stunt virus-satRNA interactions revealed by systems biology approach
Source: Plant Cell Rep. 2021 May 24;40(7):1247–67. doi: 10.1007/s00299-021-02706-4 (PMC8233301; doi:10.1007/s00299-021-02706-4)

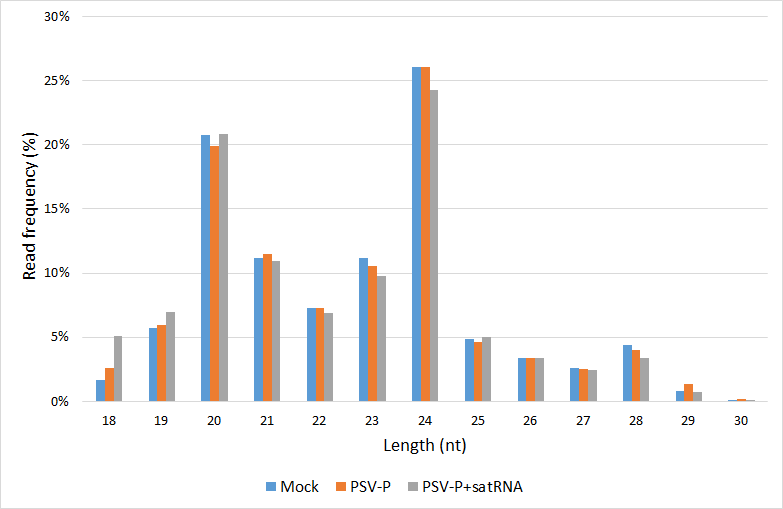

Supplement: Supplementary file 1 — Supplementary file1 Figure S1. Size distribution of the redundant sRNA sequence reads. The analysis was performed on four replicates for each condition. (TIF 22 kb) [file 299_2021_2706_MOESM1_ESM.tif]

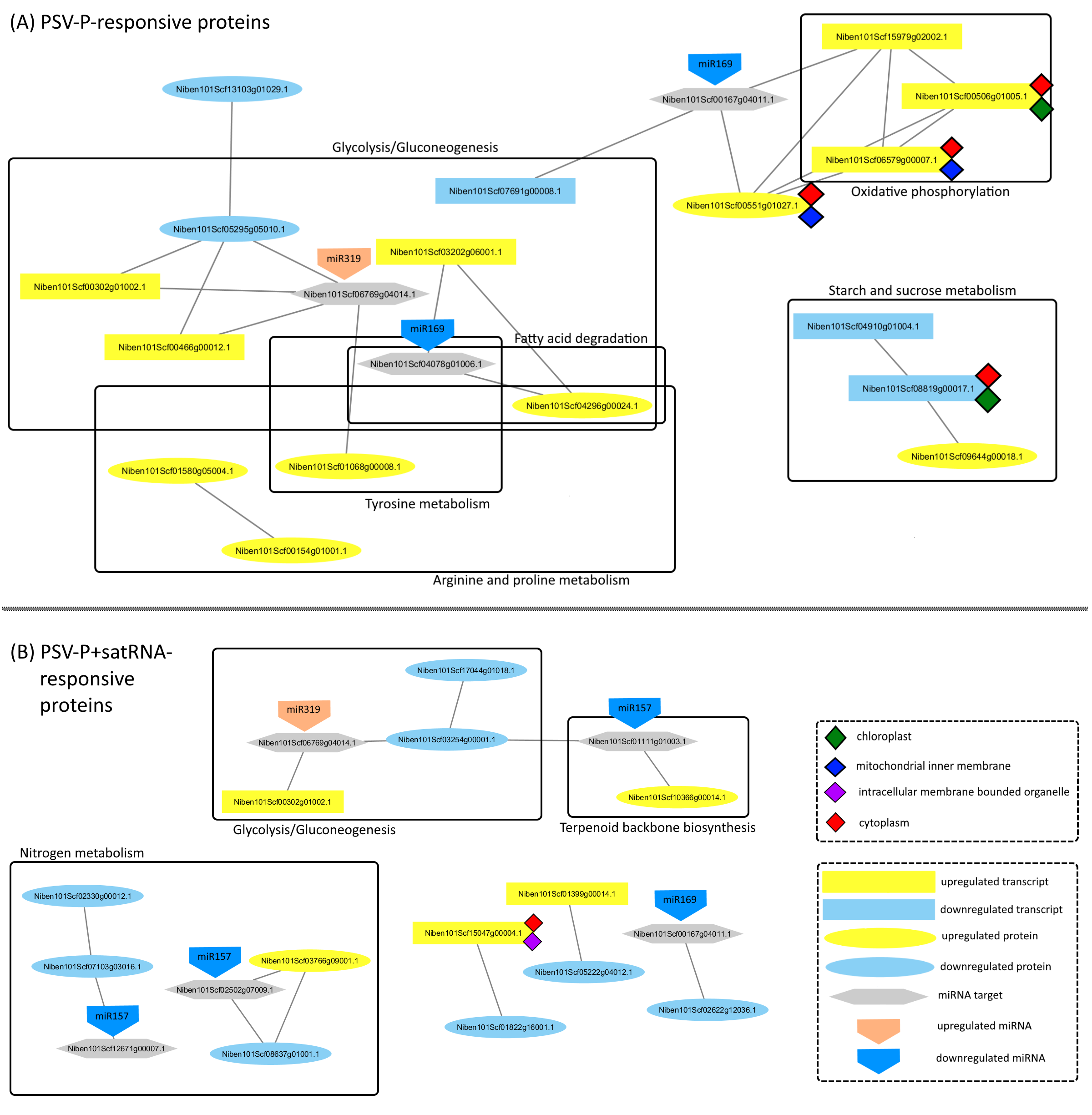

Supplement: Supplementary file 2 — Supplementary file2 Figure S2. Networks of potential protein–protein interactions of PSV-P-responsive (A) and PSV-P+satRNA-responsive (B) proteins from N. benthamiana (retrieved from proteomic, transcriptomic, and sRNA-seq analyses), differentially changed in their abundance. (TIF 684 kb) [file 299_2021_2706_MOESM2_ESM.tif]
