# Supplementary material for: Multiple cellular compartments engagement in Nicotiana benthamiana-peanut stunt virus-satRNA interactions revealed by systems biology approach
Source: Plant Cell Rep. 2021 May 24;40(7):1247–67. doi: 10.1007/s00299-021-02706-4 (PMC8233301; doi:10.1007/s00299-021-02706-4)
Supplement: Supplementary file 3 — Supplementary file3 (DOCX 16 kb) [file 299_2021_2706_MOESM3_ESM.docx]

**Multiple cellular compartments engagement in *Nicotiana benthamiana*-peanut stunt virus-satRNA interactions revealed by systems biology approach**

***Plant Cell Reports***

Barbara Wrzesińska, Agnieszka Zmienko, Lam Dai Vu, Ive De Smet, Aleksandra Obrępalska-Stęplowska*

*Corresponding author: Aleksandra Obrępalska-Stęplowska

Department of Molecular Biology & Biotechnology, Institute of Plant Protection – National Research Institute, 20 Władysława Węgorka Street, 60-318 Poznań, Poland

e-mail: olaob@o2.pl or [ao.steplowska@iorpib.poznan.pl](mailto:ao.steplowska@iorpib.poznan.pl)

tel.: +48-61-864-9145

**Table S1.** Primers used for kinase transcripts expression (that may be the targets of PSV-specific sRNAs) and the targets of miRNA measurements by RT-qPCR.

| Primer name | Primer sequence (5’-3’) | Amplicon length [bp] | Annealing temperature [°C] | Gene |
| --- | --- | --- | --- | --- |
| kinase transcripts | | | | |
| NbCLAVATA2 | F: GGGAAAAATCCATCCTCGTC | 178 | 58 | leucine-rich repeat receptor-like protein CLAVATA2 (probable) |
|  | R:ATGCTGCAAACGTGACAAAT |  |  |  |
| NbFLS2 | F: CACACTACCTTCCCCAGACC | 176 | 58 | LRR receptor-like serine/threonine protein kinase FLS2 (probable) |
|  | R: AAAGTTGAGGCTGTCAAATGG |  |  |  |
| NbGsSRK | F: GACCGAGCTGTTCCAAGAAG | 229 | 60 | G-type lectin S-receptor-like serine/threonine protein kinase At1g11330 (probable) |
|  | R: CGGATCTTTTGTCCTTGAGC |  |  |  |
| NbMAPK9 | F: CAATTTGCACATCTGGAGGA | 220 | 60 | mitogen-activated protein kinase 9 (probable) |
|  | R: TTCCGTTTTGTGTACCGACA |  |  |  |
| miRNA target transcripts | | | | |
| NbAGO1B | F: AGACAACCACTGGGTGAAGG | 152 | 60 | protein argonaute 1B (Wrzesińska, et al. 2018) |
|  | R:TTCAGAAGCTGGCTCACAAA |  |  |  |
| NbCPR5 | F: ACCTCCGACATCATGGCTAC | 193 | 56 | protein CPR-5 (probable) |
|  | R: TACCCCTTTGAGCAAACGAC |  |  |  |

Wrzesińska B, Dai Vu L, Gevaert K, De Smet I, Obrępalska-Stęplowska A (2018) Peanut stunt virus and its satellite RNA trigger changes in phosphorylation in *N. benthamiana* infected plants at the early stage of the infection. International Journal of Molecular Sciences 19:3223
