# Supplementary material for: Multiple cellular compartments engagement in Nicotiana benthamiana-peanut stunt virus-satRNA interactions revealed by systems biology approach
Source: Plant Cell Rep. 2021 May 24;40(7):1247–67. doi: 10.1007/s00299-021-02706-4 (PMC8233301; doi:10.1007/s00299-021-02706-4)
Supplement: Supplementary file 4 — Supplementary file4 (DOCX 16 kb) [file 299_2021_2706_MOESM4_ESM.docx]

**Multiple cellular compartments engagement in *Nicotiana benthamiana*-peanut stunt virus-satRNA interactions revealed by systems biology approach**

***Plant Cell Reports***

Barbara Wrzesińska, Agnieszka Zmienko, Lam Dai Vu, Ive De Smet, Aleksandra Obrępalska-Stęplowska*

*Corresponding author: Aleksandra Obrępalska-Stęplowska

Department of Molecular Biology & Biotechnology, Institute of Plant Protection – National Research Institute, 20 Władysława Węgorka Street, 60-318 Poznań, Poland

e-mail: olaob@o2.pl or [ao.steplowska@iorpib.poznan.pl](mailto:ao.steplowska@iorpib.poznan.pl)

tel.: +48-61-864-9145

**Table S2. Primers used for miRNA validation by stem-loop RT-qPCR.**

| Target sequence | Primer name | Primer sequence (5’-3’) |
| --- | --- | --- |
| miR157 | miR157RTqPCR | GTCGTATCCAGTGCAGGGTCCGAGGTATTCGCACTGGATACGACGTGCTC |
|  | miR157FwqPCR | GCTGCGCTTGACAGAAGATAGA |
| miR319 | miR319RTqPCR | GTCGTATCCAGTGCAGGGTCCGAGGTATTCGCACTGGATACGACGGGAGC |
|  | miR319FwqPCR | GCTACGTTTGGACTGAAGGGA |
| U6 | U6cDNAsynt | GTGCAGGGTCCGAGGTTTTGGACCATTTCTCGAT |
|  | U6FwqPCR | GGAACGATACAGAGAAGATTAGCA |
| Reverse primer | UniRwqPCR | GTGCAGGGTCCGAGGT |
