# Supplementary material for: Multiple cellular compartments engagement in Nicotiana benthamiana-peanut stunt virus-satRNA interactions revealed by systems biology approach
Source: Plant Cell Rep. 2021 May 24;40(7):1247–67. doi: 10.1007/s00299-021-02706-4 (PMC8233301; doi:10.1007/s00299-021-02706-4)
Supplement: Supplementary file 7 — Supplementary file7 (DOCX 17 kb) [file 299_2021_2706_MOESM7_ESM.docx]

**Multiple cellular compartments engagement in *Nicotiana benthamiana*-peanut stunt virus-satRNA interactions revealed by systems biology approach**

***Plant Cell Reports***

Barbara Wrzesińska, Agnieszka Zmienko, Lam Dai Vu, Ive De Smet, Aleksandra Obrępalska-Stęplowska*

*Corresponding author: Aleksandra Obrępalska-Stęplowska

Department of Molecular Biology & Biotechnology, Institute of Plant Protection – National Research Institute, 20 Władysława Węgorka Street, 60-318 Poznań, Poland

e-mail: olaob@o2.pl or [ao.steplowska@iorpib.poznan.pl](mailto:ao.steplowska@iorpib.poznan.pl)

tel.: +48-61-864-9145

**Table S5.** RT-qPCR validation of microarray-derived DEGs in infected *N. benthamiana* plants (yellow – up-regulated genes, blue – down-regulated genes, FC - fold change). The expression changes values in colored cells were statistically significant (p-value < 0.05).

| **Gene** | **PSV-P** | | | | **PSV-P+satRNA** | | | |
| --- | --- | --- | --- | --- | --- | --- | --- | --- |
|  | **Microarray**  **[log2 FC]** | **P-value** | **RT-qPCR**  **[log2 FC]** | **P-value** | **Microarray**  **[log2 FC]** | **P-value** | **RT-qPCR**  **[log2 FC]** | **P-value** |
| **MBF1C** | 3.999 | 0.000 | 4.863 | 0.000 | 2.707 | 0.011 | 3.464 | 0.000 |
| **PR1** | 3.676 | 0.038 | 5.004 | 0.000 | not statistically significant changed DEG | | | |
| **EF1delta** | 2.658 | 0.000 | 0.999 | 0.000 | 1.803 | 0.001 | 0.628 | 0.002 |
| **AGO2** | 2.523 | 0.000 | 1.625 | 0.000 | 2.244 | 0.000 | 1.619 | 0.000 |
| **PNO1** | 2.167 | 0.000 | 0.725 | 0.001 | 1.773 | 0.006 | 0.511 | 0.038 |
| **PR2** | 1.914 | 0.010 | 4.984 | 0.000 | not statistically significant changed DEG | | | |
| **PPCK** | -1.765 | 0.001 | -2.490 | 0.000 | -1.597 | 0.006 | -2.079 | 0.000 |
| **IRT1** | -2.258 | 0.001 | -4.028 | 0.004 | -2.000 | 0.007 | 0.306 | 0.800 |
| **PAP1** | -2.26 | 0.010 | -3.778 | 0.001 | not statistically significant changed DEG | | | |
| **ABCC** | -2.346 | 0.011 | -3.499 | 0.000 | not statistically significant changed DEG | | | |
