# Supplementary material for: Multiple cellular compartments engagement in Nicotiana benthamiana-peanut stunt virus-satRNA interactions revealed by systems biology approach
Source: Plant Cell Rep. 2021 May 24;40(7):1247–67. doi: 10.1007/s00299-021-02706-4 (PMC8233301; doi:10.1007/s00299-021-02706-4)
Supplement: Supplementary file 9 — Supplementary file9 (DOCX 16 kb) [file 299_2021_2706_MOESM9_ESM.docx]

**Multiple cellular compartments engagement in *Nicotiana benthamiana*-peanut stunt virus-satRNA interactions revealed by systems biology approach**

***Plant Cell Reports***

Barbara Wrzesińska, Agnieszka Zmienko, Lam Dai Vu, Ive De Smet, Aleksandra Obrępalska-Stęplowska*

*Corresponding author: Aleksandra Obrępalska-Stęplowska

Department of Molecular Biology & Biotechnology, Institute of Plant Protection – National Research Institute, 20 Władysława Węgorka Street, 60-318 Poznań, Poland

e-mail: olaob@o2.pl or [ao.steplowska@iorpib.poznan.pl](mailto:ao.steplowska@iorpib.poznan.pl)

tel.: +48-61-864-9145

**Table S7.** MiRNAs expression level analysis in *N. benthamiana* plants harvested at 5 dpi by means of RT-qPCR (yellow – up-regulated genes, blue – down-regulated genes, FC - fold change). The expression changes values in colored cells were statistically significant (p-value < 0.05).

| **miR** | **PSV-P** | | | | **PSV-P+satRNA** | | | |
| --- | --- | --- | --- | --- | --- | --- | --- | --- |
|  | **sRNA-seq**  **[log2 FC]** | **P-value** | **RT-qPCR**  **[log2 FC]** | **P-value** | **sRNA-seq**  **[log2 FC]** | **P-value** | **RT-qPCR**  **[log2 FC]** | **P-value** |
| **miR169** | -1.55 | 0.000 | -0.397 | 0.000 | -1.13 | 0.000 | -0.259 | 0.004 |
| **miR319** | 1.16 | 0.000 | 1.282 | 0.000 | 1.48 | 0.000 | 0.348 | 0.135 |
